# Complex molecular and functional outcomes of single versus sequential cytokine stimulation of rat microglia

**DOI:** 10.1186/s12974-016-0531-9

**Published:** 2016-03-24

**Authors:** Tamjeed A. Siddiqui, Starlee Lively, Lyanne C. Schlichter

**Affiliations:** Genes and Development Division, Krembil Research Institute, University Health Network, Toronto, Ontario M5T 2S8 Canada; Department of Physiology, University of Toronto, Toronto, Ontario Canada; Krembil Discovery Tower, Krembil Research Institute, Room 7KD-417, 60 Leonard Street, Toronto, Ontario M5T 2S8 Canada

**Keywords:** Neuroinflammation, CNS phagocytes, Microglia molecular polarization, Microglial activation states, M1, M2a, M2c activation, Myelin phagocytosis, Phagocytosis receptor expression, K^+^ channels, Ca^2+^ channels, ROS production

## Abstract

**Background:**

Microglia are the “professional” phagocytes of the CNS. Phagocytosis is crucial for normal CNS development and maintenance, but it can be either beneficial or detrimental after injury or disease. For instance, white matter damage releases myelin debris that must be cleared by microglia in order for re-myelination to occur. However, phagocytosis can also produce damaging reactive oxygen species (ROS). Furthermore, microglia can acquire pro-inflammatory (M1) or anti-inflammatory (M2) activation states that affect cell functions. Although microglia are exposed to a changing cytokine environment after injury or disease, little is known about the molecular and functional consequences. Therefore, we applied several microglial activation paradigms, with or without myelin debris. We assessed ***(i)*** gene expression changes reflecting microglial activation and inflammatory states, and receptors and enzymes related to phagocytosis and ROS production, ***(ii)*** myelin phagocytosis and production of ROS, and ***(iii)*** expression and contributions of several ion channels that are considered potential targets for regulating microglial behavior.

**Methods:**

Primary rat microglia were exposed to cytokines, individually or sequentially. First, responses to individual M1 or M2 stimuli were compared: IFN-γ plus TNF-α (“I + T”; M1 activation), interleukin-4 (M2a/alternative activation), and interleukin-10 (M2c/acquired deactivation). Second, sequential cytokine addition was used to assess microglia repolarization and cell functions. The paradigms were M2a→M1, M2c→M1, M1→M2a, and M1→M2c.

**Results:**

M1 stimulation increased pro-inflammatory genes, phagocytosis, and ROS, as well as expression of Kv1.3, KCa3.1, and Kir2.1 channels. M2a stimulation increased anti-inflammatory genes, ROS production, and Kv1.3 and KCa3.1 expression. Myelin phagocytosis enhanced the M1 profile and dampened the M2a profile, and both phagocytosis and ROS production were dependent on NOX enzymes and Kir2.1 and CRAC channels. Importantly, microglia showed some capacity for re-polarization between M1 and M2a states, based on gene expression changes, myelin phagocytosis, and ROS production.

**Conclusions:**

In response to polarizing and re-polarizing cytokine treatments, microglia display complex changes in gene transcription profiles, phagocytic capacity, NOX-mediated ROS production, and in ion channels involved in microglial activation. Because these changes might affect microglia-mediated CNS inflammation, they should be considered in future experimental, pre-clinical studies.

## Background

Phagocytosis is crucial during central nervous system (CNS) development and in the healthy adult to both promote and maintain appropriate synaptic connections and homeostasis. After CNS injury or disease, phagocytosis is crucial for clearing cellular debris, including degenerated myelin after acute injury or degenerative diseases, such as multiple sclerosis. For CNS repair to occur, myelin debris must be rapidly removed because it inhibits differentiation of oligodendrocyte precursor cells and re-myelination, and promotes formation of membrane attack complexes that further damage myelin [1–3]. Microglia are the “professional” phagocytes of the CNS. However, phagocytosis can have both beneficial and detrimental effects (reviewed in [3–9]). Microglia can exacerbate damage by producing excessive reactive oxygen species (ROS) during phagocytosis [10–12]. This ROS production, which is mediated by nicotinamide adenine dinucleotide phosphate (NADPH) oxidases (NOX enzymes), can contribute to DNA damage, lipid peroxidation, toxic protein alterations, and neurotoxicity [13–16].

In considering both harmful and helpful outcomes of phagocytosis, it is important to note that microglia can adopt several activation states or possibly a continuum of states (reviewed in [17–20]). It is generally thought that the pro-inflammatory (M1) state exacerbates tissue damage and that anti-inflammatory (M2) states help in tissue repair; however, very few studies have compared phagocytosis and ROS production in different activation states. Experimentally, M1 activation is usually evoked by lipopolysaccharide (LPS), with or without the pro-inflammatory cytokine, interferon gamma (IFN-γ). M2a (alternative) activation is evoked by interleukin-4 (with or without interleukin (IL)-13). M2c (acquired deactivation) is usually evoked by IL-10 (less often by glucocorticoid hormones). However, some suggest that IL-10 evokes gene changes more closely resembling an M1 profile [21]. Further complexity in microglial activation in vivo arises from dynamic changes in the CNS environment over time after injury or disease.

In vitro studies have addressed the microglial phagocytic capacity for plastic beads, bacteria, apoptotic cells, red blood cells, and less often, myelin. Little is known about the relationship between microglial activation states, myelin phagocytosis and ROS production, and, conversely, how myelin phagocytosis affects microglial activation states. The limited studies report that M1 activation of rat microglia increases phagocytosis of myelin (LPS or IFN-γ stimulation) [22]. Results on M2-activated microglia are inconsistent. M2a activation with IL-4 and/or IL-13 increased myelin phagocytosis by human microglia [23] but not rat microglia [22]. M2c activation of rat microglia with IL-10 increased myelin phagocytosis [22]. It is not known how sequentially exposing microglia to M1- versus M2-inducing cytokines affects their activation state, phagocytic capacity, or ROS production.

We began by comparing unstimulated, M1- and M2-activated rat microglia, quantifying myelin phagocytosis, ROS production, and expression of inflammatory mediators and receptors/enzymes related to phagocytosis and ROS production. Then, we examined how all these responses were affected by applying an M1 stimulus followed by an M2 stimulus and vice versa. Finally, under all the activation paradigms, we assessed expression and contributions of several ion channels that are known to regulate other functions of rat microglia: KCa3.1, Kv1.3, Kir2.1, as well as Orai and stromal interaction molecule (STIM) subunits that form store-operated and Ca^2+^ release-activated Ca^2+^ (CRAC) channels.

## Methods

### Primary cultures of rat microglia and activation states

Handling and sacrifice of rat pups was in accordance with guidelines from the Canadian Council on Animal Care and approved by the University Health Network Animal Care Committee (Animal Use Permit #914). Pure microglial cultures were prepared from 1–2-day-old Sprague-Dawley rat pups (Charles River, St. Constant, PQ, Canada), as before [24–27]. In brief, the meninges and cerebellum were removed from the excised brain, and the remaining tissue was mashed in cold minimal essential medium (MEM; Invitrogen, Carlsbad, CA), strained, and centrifuged at 300×*g* for 10 min. After re-suspending in MEM, cells were seeded in MEM that contained 10 % heat-inactivated fetal bovine serum (FBS; Wisent St. Bruno, PQ, Canada) and 0.05 mg/mL gentamycin (Invitrogen). The cells were incubated at 37 °C with 5 % CO_2_ for 48 h, and then, the medium was changed to remove cellular debris and non-adherent cells. After 5–6 days, microglia were harvested by shaking the flasks for 3–4 h on an orbital shaker at 70 rpm (37 °C, 5 % CO_2_). The supernatant that contained microglia was collected and centrifuged (300×*g*, 10 min), and the pellet was re-suspended in fresh MEM with 2 % heat-inactivated FBS and 0.05 mg/mL gentamycin. Microglia were seeded onto 96-well tissue culture plates at 7–8 × 10^4^ cells/well (for phagocytosis and ROS assays), 7–8 × 10^4^ cells/15 mm coverslip (for fluorescence microscopy), and >10^5^ cells/coverslip (for mRNA collection).

To induce different activation states, microglia were exposed to recombinant rat cytokines (R&D Systems Inc., Minneapolis, MN). A classical (M1) state was induced by 20 ng/mL IFN-γ plus 50 ng/mL TNF-α (for 24 h): a treatment we refer to as “I + T”. Alternative activation (M2a) was induced by 20 ng/mL IL-4 (for 24 h). Acquired deactivation (M2c) was induced with 20 ng/mL IL-10 (for 24 h). Stock solutions were made in sterile PBS with 0.3 % bovine serum albumin and stored at –20 °C. For sequential cytokine additions, the first treatment (IL-4 or I + T) was applied for 2 h, and then, the second (IL-4, I + T, IL-10) was added for an additional 22 h without washing.

### nCounter gene expression assay (NanoString)

This multiplexed assay quantifies expression of multiple, investigator-selected genes in a single ribonucleic acid (RNA) sample, with sensitivity comparable to real-time quantitative RT-PCR [28]. We collected samples from separate microglia cultures (*n* = 6) and extracted total RNA using TRIzol reagent (Invitrogen) and the RNeasy Mini Kit (QIAGEN, Mississauga, ON, Canada), as previously described [24, 25, 29]. RNA samples were stored at –80°C. Then, 200 ng of extracted RNA from each separate sample was sent for analysis to the Princess Margaret Genomics Centre (www.pmgenomics.ca; Toronto, Canada). A custom CodeSet (Table 1) was designed by NanoString Technologies (Seattle, WA) for genes we had selected based on previously reported changes with M1 and M2 activation states, their role in phagocytosis or ROS production, and the channels being investigated in this study. RNA integrity was assessed using Nanodrop 1000, and then, the nCounter Gene Expression Assay (sample hybridization, detection, scanning) was conducted according to NanoString Technologies’ recommendations. Data were normalized to the geometric mean expression of three housekeeping genes: hypoxanthine guanine phosphoribosyl transferase 1 (HPRT1), β-glucuronidase (GusB), and 60S ribosomal protein L32 (Rpl32). Reporter probe counts, which reflect the numbers of a particular mRNA transcript in the 200 ng RNA sample, were analyzed and quantified using the nCounterTM digital analyzer software2.

**Table 1:** Target sequences used to design custom CodeSet for nCounter assay

Gene	Gene accession #	Target sequence
Aif1 (Iba1)	NM_017196.2	ATCGATATTATGTCCTTGAAGCGAATGCTGGAGAAACTTGGGGTTCCCAAGACCCATCTAGAGCTGAAGAAATTAATTAGAGAGGTGTCCAGTGGCTCCG
Ccl22	NM_057203.1	TACATCCGTCACCCTCTGCCACCACGTTTCGTGAAGGAGTTCTACTGGACCTCAAAGTCCTGCCGCAAGCCTGGCGTCGTTTTGATAACCATCAAGAACC
Cd11b (Itgam)	NM_012711.1	CATCCCTTCCTTCAACAGTAAAGAAATATTCAACGTCACCCTCCAGGGCAATCTGCTATTTGACTGGTACATCGAGACTTCTCATGACCACCTCCTGCTT
Cd68 (ED1)	NM_001031638.1	CTCTCATTCCCTTACGGACAGCTTACCTTTGGATTCAAACAGGACCGACATCAGAGCCACAGTACAGTCTACCTTAACTACATGGCAGTGGAATACAATG
Cd163	NM_001107887.1	CCTCTGTAATTTGCTCAGGAAACCAATCGCATACACTGTTGCCATGTAGTTCATCATCTTCGGTCCAAACAACAAGTTCTACCATTGCAAAGGACAGTGA
C1r	XM_001061611.1	ACAAAGACCTTATGGGTTATGTCAGCGGCTTCGGGATAACAGAAGATAAAATAGCTTTTAATCTCAGGTTTGTCCGTCTGCCCATAGCCGATCGAGAGGC
Cybb (Nox2)	NM_023965.1	CAGTACCAAAGTTTGCCGGAAACCCTCCTATGACTTGGAAATGGATCGTGGGTCCCATGTTCCTGTATCTGTGTGAGAGGCTGGTGCGGTTTTGGCGATC
Ptgs2 (COX-2)	NM_017232.3	TTCGGAGGAGAAGTGGGTTTTAGGATCATCAACACTGCCTCAATTCAGTCTCTCATCTGCAATAATGTGAAAGGGTGTCCCTTTGCCTCTTTCAATGTGC
Cx3cr1	NM_133534.1	ATGTGCAAGCTCACGACTGCTTTCTTCTTCATTGGCTTCTTTGGGGGCATATTCTTCATCACCGTCATCAGCATCGACCGGTACCTCGCCATCGTCCTGG
FcγR1a	NM_001100836.1	TGATGGATCATACTGGTGCGAGGTAGCCACGGAGGACGGCCGTGTCCTTAAGCGCAGCACCAAGTTGGAGCTATTTGGTCCCCAGTCATCAGATCCTGTC
FcγR2b	NM_175756.1	CTGGTCCAAGGAATGCTGTAGATATGAAAGAAAACATCTAGAGTCCCTTCTGTGAGTCCTGAAACCAACAGACACTACGATATTGGTTCCCAATGGTTGA
FcγR3a	NM_207603.1	GACTCTTGTTTGCAATAGACACAGTGCTGTATTTCTCGGTGCAGAGGAGTCTTCAAAGTTCCGTGGCAGTCTATGAGGAACCCAAACTTCACTGGAGCAA
Gusb	NM_017015.2	TCATTTGATCCTGGATGAGAAACGAAAAGAATATGTCATCGGAGAGCTCATCTGGAATTTTGCTGACTTCATGACGAACCAGTCACCACTGAGAGTAACA
Havcr2 (TIM-3)	NM_001100762.1	CGATGAAATTAAGGACTCTGGAGAAACTATCAGAACTGCTGTCCACATTGGAGTAGGCGTCTCTGCTGGGCTGGCCCTGGCACTTATTCTTGGTGTTTTA
Hprt1	NM_012583.2	AGCTTCCTCCTCAGACCGCTTTTCCCGCGAGCCGACCGGTTCTGTCATGTCGACCCTCAGTCCCAGCGTCGTGATTAGTGATGATGAACCAGGTTATGAC
Hvcn1 (Hv1)	XM_006249369.2	ACCAAGAGGATGAGCAGGTTCTTGAAGCACTTCACAGTGGTGGGGGACGACTACCACACCTGGAATGTCAACTACAAGAAGTGGGAGAACGAGGAGGATG
Il6	NM_012589.1	GGAACAGCTATGAAGTTTCTCTCCGCAAGAGACTTCCAGCCAGTTGCCTTCTTGGGACTGATGTTGTTGACAGCCACTGCCTTCCCTACTTCACAAGTCC
Kcna3 (Kv1.3)	NM_019270.3	GCCACCTTCTCCAGAAATATCATGAACCTGATAGACATTGTAGCCATCATCCCTTATTTTATTACTCTGGGCACTGAGCTGGCTGAGCGACAGGGTAATG
Kcnj2 (Kir2.1)	NM_017296.1	GTTCTTTGGCTGTGTGTTTTGGTTGATAGCTCTGCTCCACGGGGATCTGGATGCTTCTAAAGAGAGCAAAGCGTGTGTGTCTGAGGTCAACAGCTTCACG
Kcnn4 (KCa3.1)	NM_023021.2	TACGTCTCTACCTGGTGCCTCGCGCGGTACTTCTGCGTAGCGGGGTCCTGCTCAACGCGTCTTACCGCAGCATCGGGGCGCTCAACCAAGTCCGATTCCG
Mrc1	NM_001106123.1	CTTTGGAATCAAGGGCACAGAGCTATATTTTAACTATGGCAACAGGCAAGAAAAGAATATCAAGCTTTACAAAGGTTCCGGTTTGTGGAGCAGATGGAAG
Msr1 (SR-A)	NM_001191939.1	CACGTTCCATGACAGCATCCCTTCCTCACAACACTATAAATGGCTCCTCCGTTCAGGAGAAACTGAAGTCCTTCAAAGTTGCCCTCGTCGCTCTCTACCT
Myc	NM_012603.2	ACCGAGGAAAACGACAAGAGGCGGACACACAACGTCTTGGAACGTCAGAGGAGAAACGAGCTGAAGCGTAGCTTTTTTGCCCTGCGCGACCAGATCCCTG
Ncf1	NM_053734.2	TCCATTCCCAGCATCCCATAATTGGGCTTGTCCGTGTTCCAACATCTGGGCGGAATTTCACAGCCAAAGGTCAAGAGGACTGCTGTTACGTTCAAGGTCG
Nos2 (iNOS)	NM_012611.2	ACGGGACACAGTGTCGCTGGTTTGAAACTTCTCAGCCACCTTGGTGAGGGGACTGGACTTTTAGAGACGCTTCTGAGGTTCCTCAGGCTTGGGTCTTGTT
Nox1	NM_053683.1	CCGAGAAAGAAGATTCTTGGCTAAATCCCATCCAGTCTCCAAACGTGACAGTGATGTATGCAGCATTTACCAGTATTGCTGGCCTTACTGGAGTGGTCGC
Nox4	NM_053524.1	TGTTGGACAAAAGCAAGACTCTACATATCACCTGTGGCATAACTATTTGTATTTTCTCAGGTGTGCATGTAGCTGCCCACTTGGTGAACGCCCTGAACTT
P2ry6	NM_057124.2	TGGCCCAACATGCCTGGCCCTCCAAAATTTCTATGTCAACCACAAAACTAAGACACCTGTGTTTCGGGGACTGGTCAGTTCATGCTTGTTATACCAGAAT
Orai1	NM_001013982.1	GCCTTCTCCACCGTCATCGGGACGCTGCTTTTCCTGGCCGAAGTCGTGCTGCTCTGCTGGGTGAAGTTCTTACCGCTCAAGAGGCAGGCGGGACAGCCAA
Orai3	NM_001014024.1	ACCTGTAATGTGCTTTACAGTTGGCATCCTGGGAGAGATTTTACATAGGCTCCTCAGATGAACCACTTTACACTTGGTGACTTGTGGTGGTGTGTCCCAC
Rpl32	NM_013226.2	CATCGTAGAAAGAGCAGCACAGCTGGCCATCAGAGTCACCAATCCCAACGCCAGGCTACGCAGCGAAGAGAATGAATAGATGGCTTGTGTGCCTGTTTTG
Sirpa	NM_013016.2	AGGACATTCATTCTCGGGTCATCTGCGAGGTAGCCCACGTCACCTTGGAAGGACGCCCGCTTAATGGGACCGCTAACTTTTCTAACATCATCCGAGTTTC
Stim1	NM_001108496.2	TATCTATCGTGATTGGTGTGGGTGGCTGCTGGTTTGCCTATATCCAGAACCGTTACTCTAAGGAGCACATGAAGAAAATGATGAAGGATCTGGAAGGATT
Stim2	NM_001105750.2	TTCACAATTGGACGCTTGAGGATACCCTGCAGTGGTTGATAGAATTTGTTGAACTCCCACAATACGAGAAGAATTTTAGGGATAATAATGTGAAAGGAAC
Tnfa	NM_012675.2	GGTGATCGGTCCCAACAAGGAGGAGAAGTTCCCAAATGGGCTCCCTCTCATCAGTTCCATGGCCCAGACCCTCACACTCAGATCATCTTCTCAAAACTCG
Trem2	NM_001106884.1	TCCGGCTGGCTGAGGAAGGGTGCCATGGAACCTCTCCACGTGTTTGTCCTGTTGCTGGTCACAGAGCTGTCCCAAGCCCTCAACACCACAGTGCTGCAGG

### Myelin phagocytosis and ROS production

Myelin was isolated by homogenizing adult rat brains (weighing 1.95–2.10 g) in iso-osmotic buffer, followed by sucrose gradient centrifugation [30]. The myelin concentration was calculated from the total protein concentration, using the Pierce bicinchoninic acid (BCA) assay (Thermo Fisher Scientific, Waltham, MA), and adjusted as required. Myelin was labeled in the dark with the lipophilic dye, 1,1′-dioctadecyl-3,3,3′,3′-tetramethyl-indocarbocyanine perchlorate (DiI) (Molecular Probes, Burlington, ON, Canada) (1:200; ≥2 min, 37 °C), as is commonly done for phagocytosis studies [10, 31]. Microglia were seeded at 7–8 × 10^4^ cells per 15-mm glass coverslip. They were cultured for 1–2 days without complement proteins [32] by using 2 % heat-inactivated FBS at 37 °C, 5 % CO_2_, and then incubated with DiI-labeled myelin. Extracellular myelin was removed by washing with standard bath solution (for phagocytosis and ROS assays), Trizol reagent (for RNA isolation) or fixative (for fluorescence microscopy). The cells were plated at the same high density, and the plate reader was configured to measure fluorescence intensity at the bottom of the plate. At the end of every experiment, we monitored the cell health and saw no evidence of damage, death, or obvious differences in cell numbers.

Myelin phagocytosis was first optimized by incubating microglia with 5, 25, or 50 μg/mL myelin for 6 h or with 25 μg/mL myelin for 1, 3, or 6 h. Phagocytosis of DiI-labeled myelin fragments was verified by fluorescence microscopy in microglia that were fixed in 4 % paraformaldehyde (10 min, room temperature) and stained with FITC-conjugated tomato lectin (1:500, 15 min; Sigma-Aldrich, Oakville, ON) and the nuclear stain, 4′,6-diamidino-2-phenylindole (DAPI; 1:3000, 5 min; Cayman Chemical, Ann Arbor, Michigan). After washing (three times, 5 min), cells on coverslips were mounted on glass slides with Dako mounting medium (Dako, Glostrup, Denmark) and stored in the dark at 4 °C until images were acquired with an Axioplan 2 wide-field epifluorescence microscope equipped with an Axiocam HR digital camera (both from Zeiss, Toronto, ON, Canada).

**Fig. 1:**
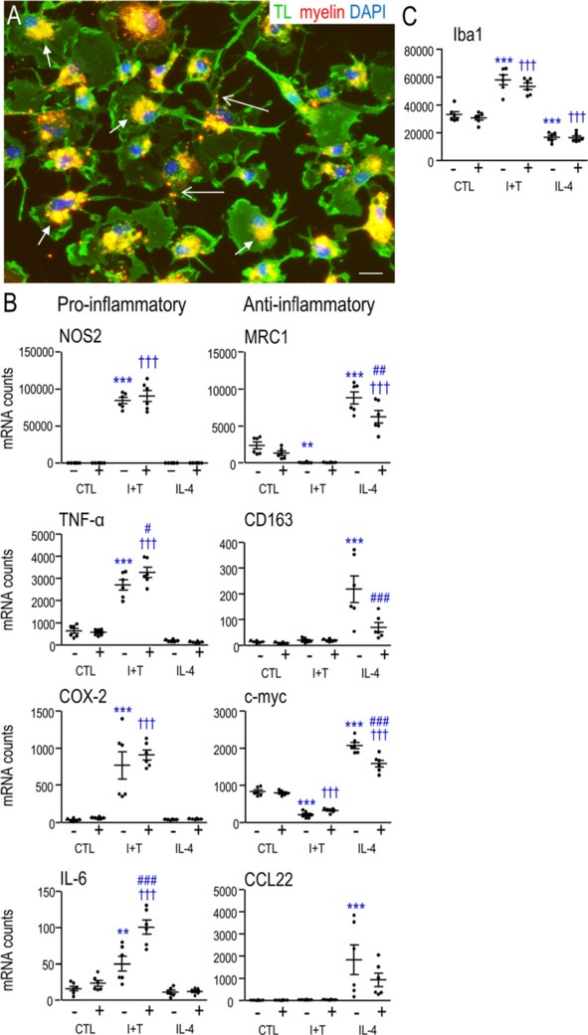
Effects of microglial activation state and exposure to myelin on inflammatory gene expression. **a** Myelin phagocytosis. Representative images showing myelin fragments inside primary rat microglia at 6 h after adding 25 μg/mL DiI-labeled myelin (*red*). Microglia were co-labeled with tomato lectin (*TL*, *green*) and the nuclear stain, *DAPI* (*blue*). Scale bar, 10 μm. *Short arrows* show peri-nuclear accumulation of myelin; *long arrows* show myelin in other cell regions. **b**, **c** Gene expression of inflammatory markers (**b**) and “ionized Ca^2+^ binding adapter molecule 1” (**c** Iba1; also known as AIF-1). Microglia were unstimulated (control, *CTL*) or stimulated for 24 h with 20 ng/mL IFN-γ and 50 ng/mL TNF-α (I + T) or 20 ng/mL IL-4, with or without a subsequent 6-h exposure to 25 μg/mL myelin (30 h total time after cytokine addition; *plus* or *minus sign* indicates presence/absence of myelin). Expression of each gene is shown as normalized mRNA counts (described in the “Methods” section). On the scatterplots, the mean ± SEM is indicated for six different microglia cultures. Data were analyzed by two-way ANOVA with Bonferroni’s post hoc test. The comparisons are as follows. *Asterisk* Between unstimulated microglia (*CTL*) and cells treated with I + T or IL-4. *Dagger sign* CTL versus activated cells in the presence of myelin. *Number sign* Effects of myelin within a particular activation state. *One symbol p* < 0.05, *two symbols p* < 0.01, *three symbols p* < 0.001

An earlier study of murine microglia showed that myelin debris was internalized within 1 h and accumulated in LAMP-1-positive lysosomes, presumably targeted for degradation [10]. We verified that unstimulated (control) rat microglia rapidly phagocytose myelin fragments under the present conditions. At 25 μg/mL myelin, uptake was substantial by 3 h, and by 6 h, DiI-labeled myelin fragments had accumulated mainly in the perinuclear region, with a small amount in other cell regions (Fig. 1a). For the remaining study, we chose a 6-h incubation with 25 μg/mL myelin. The washing procedure effectively removed extracellular myelin debris (Fig. 1a); thus, we could use a multi-label fluorescence plate reader to quantify myelin phagocytosis.

To quantify ROS, microglia were incubated (1 h, 37 °C, 5 % CO_2_) with the membrane-permeant probe, a chloromethyl derivative of 2′,7′-dichlorodihydrofluorescein diacetate (CM-H_2_DCFDA; 5 μM; Invitrogen). The probe is cleaved by intracellular esterases to release H_2_DCFDA, which is then oxidized to a fluorescent compound, dichlorodihydrofluorescein (DCF) that remains trapped in the cells. DCF (495 nm excitation, 525 nm emission) is a general ROS probe that is compatible with DiI-labeled myelin (553 nm excitation, 570 nm emission), thus allowing myelin phagocytosis and ROS production to be monitored in the same samples. Extracellular CM-H_2_DCFDA and myelin fragments were removed by washing the coverslips with standard bath solution, which contained (in mM) 125 NaCl, 5 KCl, 1 CaCl_2_, 1 MgCl_2_, 10 HEPES, and 5 D-glucose (pH 7.4; 290–300 mOsm). As a positive control for ROS production, microglia were occasionally stimulated for 1 h with the PKC activator, phorbol 12-myristate 13-acetate (PMA; 100 nM, Sigma), which produced a robust DCF signal (not shown).

To assess contributions of the ion channels, we used well-known blockers at concentrations that we previously validated are sufficient to block the channels in primary rat microglia. As before, there was no toxicity at the concentrations used. KCa3.1 (also known as IK1, SK4) was blocked with 1-[(2-chlorophenyl) diphenylmethyl]-1H-pyrazole (TRAM-34; 1 μM; Sigma) as before [24, 27, 33, 34]. Kir2.1 was blocked with *N*-[(4-methoxyphenyl)methyl]-1-naphthalene-methanamine hydrochloride (ML133; 20 μM; Tocris Bioscience, MO) as in our recent study [35]. Kv1.3 was blocked with 5-nM agitoxin-2 (Sigma) as before [36–38]. CRAC channels were blocked with *N*-[4-[3,5-bis(trifluoromethyl)-1H-pyrazol-1-yl]phenyl]-4-methyl-1,2,3-thiadiazole-5-carboxamide (BTP2; 10 μM; EMD Millipore, San Diego, CA). We previously showed that 10 μM BTP2 blocks Ca^2+^ signaling through CRAC [33, 35]. Stock solutions were made in sterile double distilled water (agitoxin-2) or DMSO (TRAM-34, ML133, BTP2) and stored at –20 °C. When used, the channel blockers were applied together with myelin.

### Statistical analysis

All graphical data are presented as mean ± standard error of the mean (SEM) for the *n* values indicated. The statistical significance of results was analyzed with a one- or two-way analysis of variance (ANOVA) and post hoc analysis as indicated in the figure legends. Results were considered significant if *p* < 0.05

## Results

### Myelin phagocytosis affects the activation state of primary rat microglia

When unstimulated (control) microglia were exposed to 25 μg/mL of myelin debris for 6 h, myelin was seen mainly in the perinuclear region, with a small amount in other cell regions (Fig. 1a). We then examined expression of over two dozen genes to create a profile of microglial responses to the activating stimuli and to myelin debris. Previously, by assessing several well-known inflammatory genes, we found that unstimulated rat microglia were in a relatively resting state [25, 29, 39]. This was confirmed, based on very low expression of well-established pro-inflammatory (M1) molecules (NOS2/iNOS, TNF-α, COX-2, IL-6), and of some markers of ‘alternative’ (M2) activation (cluster of differentiation (CD)163, CCL22). Two M2-associated molecules, c-myc and MRC1, were expressed at moderate levels, and MRC1 expression was similar to our recent study [24]. When unstimulated cells were allowed to phagocytose myelin for 6 h, none of these markers changed significantly (Fig. 1b).

The same inflammatory markers were quantified after microglia were treated with cytokines to evoke two different activation states, and again, after myelin phagocytosis (Fig. 1b, c). Previously validated M1 markers included increased NOS2, COX-2, IL-6, and a loss of M2a markers [21]. Here, classical (M1) activation was evoked by 24 h treatment with a combination of IFN-γ and TNF-α (which we call “I + T”). As expected, there was a dramatic increase in expression of pro-inflammatory molecules (NOS2, TNF-α, COX-2); IL-6 increased to a lesser degree. Conversely, IL-4 (M2a, alternative activation) increased expression of several M2-associated molecules (MRC1, CD163, c-myc, CCL22). Interestingly, while Iba1 expression increased in the M1 state and decreased in the M2 state, it was robustly expressed under all conditions (Fig. 1c) and is thus not strictly an activation marker.

Microglia were treated with cytokines (as above), exposed to myelin for a further 6 h, and gene expression was again quantified. After myelin phagocytosis, M1-polarized microglia showed increased expression of some pro-inflammatory molecules (TNF-α, IL-6). In M2a-polarized microglia, phagocytosis decreased several genes associated with alternative activation (MRC1, CD163, c-myc). Overall, these results suggest that myelin can rapidly exacerbate some pro-inflammatory responses of M1-polarized microglia and reduce M2a polarization.

### The microglial activation state affects myelin phagocytosis and expression of phagocytosis-related molecules

**Fig. 2:**
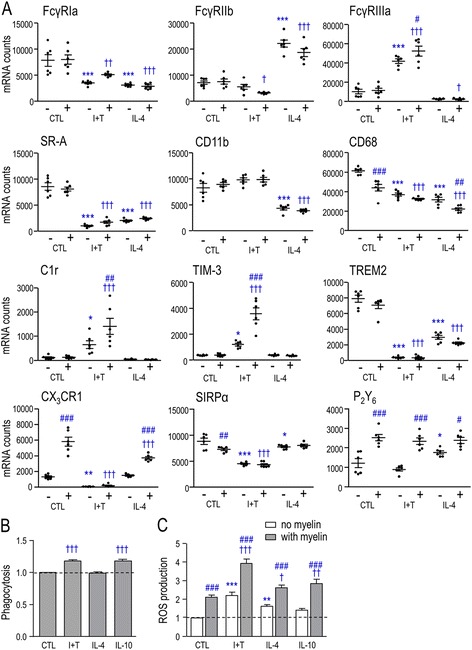
Effects of microglial activation state on expression of phagocytosis-related molecules, phagocytosis, and ROS production. **a** Phagocytosis-related genes. Microglia were unstimulated (control, *CTL*) or stimulated for 24 h with 20 ng/mL IFN-γ + 50 ng/mL TNF-α (M1 activation state) or 20 ng/mL IL-4 (M2a state), with or without a subsequent 6-h exposure to 25 μg/mL myelin (*plus* or *minus sign* indicates presence/absence of myelin), as in Fig. 1a. Values are expressed as normalized mRNA counts (described in the “Methods” section), mean ± SEM (six different cell cultures), and were analyzed by two-way ANOVA with Bonferroni’s post hoc test. **b** Phagocytosis of myelin fragments. Microglia were exposed for 6 h to 25 μg/mL DiI-labeled myelin, and the amount of internalized myelin was determined in unstimulated (CTL), M1 (I + T), M2a (IL-4), and M2c (20 ng/mL IL-10)-stimulated microglia. Results were normalized to control microglia (*dashed line*) to determine activation state-dependent changes. Data are expressed as mean ± SEM (20 individual cultures) and were analyzed by one-way ANOVA with Dunnett’s post hoc test. **c** Production of reactive oxygen species (*ROS*). Intracellular ROS was monitored with the general ROS probe, dichlorofluorescein (DCF), and normalized to DCF levels in unstimulated (*CTL*) microglia without myelin (*dashed line*). Data were analyzed by two-way ANOVA with Bonferroni’s post hoc test (*n* = 19 individual cultures). The comparisons are as follows. *Asterisk* Between unstimulated microglia (CTL) and cells treated with I + T, IL-4, or IL-10. *Dagger sign* CTL versus different activation states in the presence of myelin. *Number sign* Effects of myelin within a particular activation state. *One symbol p* < 0.05, *two symbols p* < 0.01, *three symbols p* < 0.001

It is very difficult to quantify phagocytosis in vivo; thus, many studies use surrogate phagocytosis “markers” such as the glycoprotein, CD68 (ED1) [3]. Here, we compared expression of a panel of phagocytosis-related molecules in untreated, M1- and M2a-activated microglia (Fig. 2a). The molecules were chosen for the following reasons. To phagocytose myelin in vitro, microglia primarily use the scavenger class A receptor (SR-A), complement receptor 3 (CR3) (also called α_M_β_2_ and comprised of CD11b and CD18), and immunoglobulin Fc gamma receptors (if anti-myelin antibodies are present) [2, 3, 40]. We quantified expression of SR-A, FcγRIa, FcγRIIb, FcγRIIIa, and CD11b. FcγRIa (CD64) and FcγRIIIa (CD16) stimulate phagocytosis; whereas, FcγRIIb (CD32) is inhibitory [41], as is signal regulatory protein alpha (SIRPα) (CD172a), which interacts with CD47 ligand on myelin [1]. CD68 and CR3 are often used as general markers of microglial activation because their staining intensity increases after CNS injury [7, 42–44]. C1r is an essential protease that initiates the classical complement pathway to opsonize particles with complement proteins for targeted phagocytosis [45]. Nucleotides released by damaged cells act as “find-me” signals for phagocytes, and UDP acts on metabotropic P_2_Y_6_ receptors to facilitate phagocytosis. This receptor is up-regulated in microglia in response to dying neurons [7, 46]. Recently, triggering receptor expressed on myeloid cells 2 (TREM2), CX_3_CR1, and T-cell immunoglobulin and mucin-domain containing 3 (TIM-3) have been added to the list of receptors involved in microglial phagocytosis. TREM2 senses lipid components of damaged myelin, is required for debris clearance, and is mainly expressed on microglia [47]. In the CNS, CX_3_CR1 is expressed exclusively by microglia and perivascular macrophages [48] and is required for effective clearance of myelin debris [49]. TIM-3 is present in human microglia that are specifically localized to white matter [50]. LPS increases TIM-3 expression in murine microglia, and blocking its activity decreases their phagocytosis of apoptotic neurons [51].

***(i)*** In untreated microglia, there was substantial expression (arbitrary cutoff, >5000 mRNA counts/200 ng RNA) of several stimulatory receptors (CD11b, SR-A, FcγRIa, FcγRIIIa, CD68, TREM2) and the inhibitory receptors, FcγRIIb and SIRPα (Fig. 2a). There was lower expression of C1r, P_2_Y_6_, CX_3_CR1, and TIM-3. ***(ii)*** Following I + T (M1) treatment, three stimulatory receptors increased (C1r, FcγRIIIa, TIM-3) and the inhibitory receptor, SIRPα, decreased. While this might mean that M1 cells have a higher phagocytic capacity for a wider range of targets, there were concomitant decreases in the stimulatory receptors, SR-A, FcγRIa, CD68, TREM2, and CX_3_CR1. ***(iii)*** After IL-4 treatment (M2a state), there were decreases in several stimulatory receptors (CD11b, SR-A, FcγRIa, CD68, TREM2) and an increase in the inhibitory receptor, FcγRIIb, suggesting a reduced phagocytic capacity. However, P_2_Y_6_ increased and the inhibitory receptor, SIRPα decreased. Surprisingly, both M1 and M2a activation decreased SR-A, FcγRIa, CD68, TREM2, and SIRPα.

The complex changes in expression of phagocytosis-related receptors made predictions difficult. Thus, it was important to next assess whether myelin phagocytosis was affected by the activation state and, conversely, whether myelin altered receptor expression. ***(i)*** In unstimulated microglia, many receptors were unaffected by myelin (CD11b, SR-A, FcγRIa, FcγRIIb, FcγRIIIa, C1r, TREM2) or slightly decreased (CD68, SIRPα). However, there were increases in three receptors not known to mediate myelin phagocytosis: P_2_Y_6_, CX_3_CR1, and TIM-3. ***(ii)*** In I + T-treated cells, myelin increased the stimulatory receptors, P_2_Y_6_ and TIM-3 and, to a lesser extent, FcγRIIIa and C1r. None were decreased. ***(iii)*** In IL-4-treated cells, myelin increased P_2_Y_6_ and CX_3_CR1, and although CD68 was slightly decreased, its mRNA counts remained very high. Interestingly, P_2_Y_6_ increased under all three activation states. Overall, effects of myelin were modest. ***(iv)*** Phagocytosis was unchanged by IL-4 and increased ~20 % by I + T (Fig. 2b). Here, we also tested IL-10 to represent an M2c phenotype that is thought to resolve pro-inflammatory states (reviewed in [18, 19]). We did not examine the molecular profile after IL-10 alone, mainly because the markers are less clear and some overlap with M1 markers [21]; however the IL-10-evoked increase in phagocytosis was similar to I + T. These small differences in phagocytosis are very unlikely to account for large differences in gene expression after exposure to myelin (Fig. 2a).

### The microglial activation state affects production of ROS and expression of ROS-related molecules

Phagocytosis is often accompanied by a considerable ROS production. Thus, we next compared ROS production in unstimulated (control) microglia and after I + T, IL-4, or IL-10, with and without myelin phagocytosis (Fig. 2c). Without myelin, ROS production increased in M1 (I + T) and M2a (IL-4) states. Myelin phagocytosis further increased ROS production in all activation states.

Based on these changes in ROS production, we examined expression of several molecules related to ROS production. NADPH oxidase enzymes (NOX1–5) are homologs of NOX2/gp91phox, the catalytic subunit that is present in cell membranes [52]. It was previously reported that primary rat microglia express NOX1, NOX2, and NOX4, while NOX3 was not detected [53], and our results corroborate this (we did not examine NOX3). NOX2 is well-studied and is largely responsible for the phagocytosis-induced ROS production (respiratory burst) of microglia [4, 54] and other phagocytes [55]. Activation of NOX2 at the plasma membrane requires phosphorylation of the accessory subunit, Ncf1 (neutrophil cytosolic factor 1/p47phox) [56, 57].

**Fig. 3:**
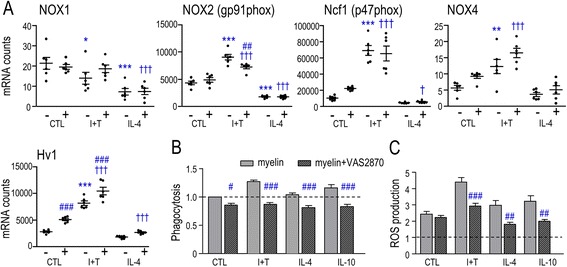
Expression of ROS-related molecules and contribution of NOX enzymes to myelin phagocytosis and ROS production. **a** Expression of ROS-related molecules. Rat microglia were stimulated with cytokines for 24 h with or without a subsequent 6-h exposure to myelin (*plus* or *minus sign* indicates presence/absence of myelin), as in Figs. 1a and 2a. **b** Effect of NOX inhibition on phagocytosis of myelin fragments. The activation treatments, assay, and normalization of data were the same as Fig. 2b, but now comparing the pan-NOX inhibitor, 5 μM VAS2870. **c** Effect of NOX inhibition on ROS levels in microglia that were treated as in Fig. 2b. As above, ROS levels were normalized to unstimulated microglia without myelin (*dashed line*), data are expressed as mean ± SEM (*n* = 6 individual cultures), and results were analyzed by two-way ANOVA with Bonferroni’s post hoc test. The comparisons are as follows: *asterisk* unstimulated (CTL) versus different activation states in the absence of myelin, *dagger sign* unstimulated (CTL) versus different activation states in the presence of myelin, *number sign* effects of myelin (**a**) or VAS2870 (**b**, **c**) within a particular activation state. *One symbol p* < 0.05, *two symbols p* < 0.01, *three symbols p* < 0.001

NOX2 and Ncf1 were highly expressed in unstimulated rat microglia, increased in M1-activated cells, and decreased in the M2a state (Fig. 3a). Myelin phagocytosis had minor effects, slightly decreasing NOX2 in the M1 state only. Our results are consistent with an earlier study of IFN-γ-stimulated microglia showing that myelin phagocytosis increased ROS production without affecting Ncf1 transcription [10]. NOX4 is unique in several respects. Its contribution is regulated by transcription without accessory subunits [58, 59], and it is located in membranes of the ER, nuclear envelope, and mitochondria [60]. We found that NOX4 expression was extremely low in unstimulated rat microglia and increased slightly in the M1 state only. NOX1 can contribute to production of both ROS and reactive nitrogen species [13], which are both involved in eradicating pathogens [55]. We found that NOX1 expression was always very low, was further decreased after I + T or IL-4 treatment, and was not affected by myelin phagocytosis. Thus, it seems most likely that NOX2 was responsible for the observed changes in ROS production (Fig. 2). The voltage-gated proton channel, Hv1, was also examined because it can facilitate ROS production by allowing H^+^ efflux as charge compensation for NOX-generated electrons [61, 62]. Hv1 was moderately expressed in unstimulated microglia and increased in the M1 state. Overall, only the M1 stimulus increased known facilitators of ROS production in microglia. Myelin phagocytosis further increased Hv1 in unstimulated and M1-activated cells.

We then used the pan-NOX inhibitor VAS2870 [63] to assess overall contributions of NOX enzymes. The drug was non-toxic. In control cells and in all three activation states, VAS2870 decreased phagocytosis to below the level of unstimulated microglia (Fig. 3b). Exposure to myelin always increased ROS production (as in Fig. 2c), and this was essentially abolished by VAS2870 in all three activated states (Fig. 3c). (Statistical comparisons of phagocytosis in different activation states were shown in Fig. 2.)

### Attempting to repolarize microglia using sequential cytokine stimulation

**Fig. 4:**
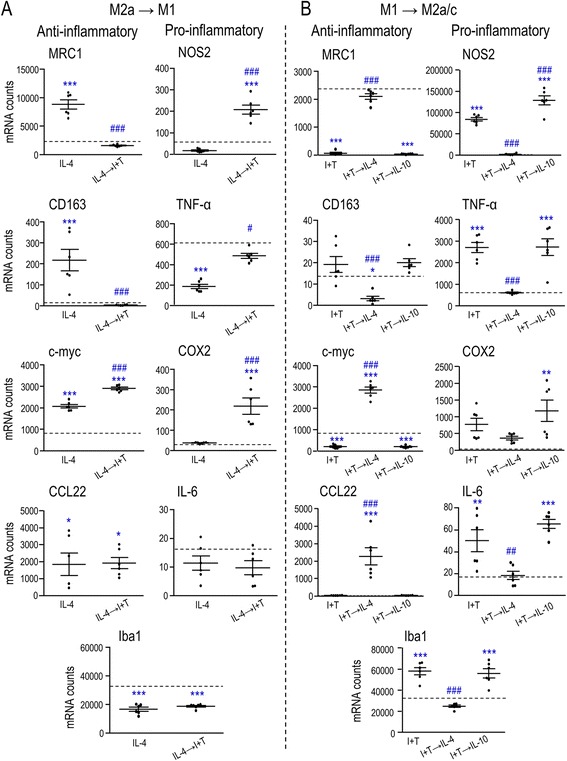
Repolarizing the inflammatory profile of microglia using sequential cytokine addition. **a** “M2a→M1” stimulus paradigm. Microglia were treated with 20 ng/mL IL-4 for 2 h followed by 20 ng/mL IFN-γ + 50 ng/mL TNF-α (I + T) for 22 h. **b** “M1→M2” stimulus paradigm. Microglia were treated with I + T for 2 h followed by either IL-4 (“M1→M2a”) or 20 ng/mL IL-10 (“M1→M2c”) for 22 h. Data are expressed as mRNA counts in 200 ng total RNA (mean ± SEM, *n* = 6 individual cultures) and were analyzed by one-way ANOVA with Tukey’s post hoc test. The *dashed lines* indicate expression levels in unstimulated (control) microglia. (Some *lines* are too low to see.) The comparisons are as follows: *asterisk* differences from control microglia, *number sign* effects of a secondary stimulus on the first stimulus. *One symbol p* < 0.05, *two symbols p* < 0.01, *three symbols p* < 0.001

To model a changing inflammatory environment, such as can occur after CNS injury (see Background), we used sequential addition of cytokines that induce M1 then M2 activation and vice versa. The same panel of inflammatory, phagocytosis-related, and oxidative stress-related genes was assessed as in Figs. 1, 2, and 3. ***(i)*** Microglia were stimulated with IL-4 followed by I + T, which we refer to as an “M2a→M1” stimulus paradigm (Fig. 4a). Several changes were consistent with I + T skewing (re-polarizing) them toward an M1 state. Two M2 markers (MRC1, CD163) were dramatically reduced (to control levels), and three pro-inflammatory mediators (NOS2, TNF-α, COX2) were higher than with IL-4 alone. The cells were not fully re-polarized to M1, as some pro-inflammatory genes remained lower than with I + T alone: NOS2/iNOS (a 3.6-fold increase vs 1444 fold), Iba1 (0.6 vs 1.8 fold), TNF-α (0.8 vs 4.3 fold), and COX-2 (7.1 vs 25.9 fold). Not all genes were affected. The changes in IL-6 and CCL22 were not different from IL-4 alone. While c-myc was increased in the M2a→M1 stimulus paradigm, the change was small (3.6 vs 2.5 fold). ***(ii)*** In the reverse paradigm, the cells were first stimulated with I + T, followed by IL-4 or IL-10 (Fig. 4b). Secondary IL-4 treatment (M1→M2a paradigm) generally dampened the M1 response and further skewed microglia toward an anti-inflammatory M2a state relative to I + T alone. That is, IL-4 prevented induction of Iba1 and down-regulated several pro-inflammatory molecules; NOS2 (5.9 vs 1444 fold), TNF-α (1.0 vs 4.3 fold), and IL-6 (1.1 vs 3.1 fold). It also increased expression of some M2a-associated molecules; CCL22 (151 vs 2.1 fold), c-myc (3.5 vs 0.2 fold), and MRC1 (0.9 vs 0.03 fold). Overall, some re-polarization of the activation state was evident between M1 and M2a. In contrast, secondary IL-10 treatment (M1→M2c paradigm) failed to reverse any I + T-induced changes, and instead, it increased expression of NOS2.

**Fig. 5:**
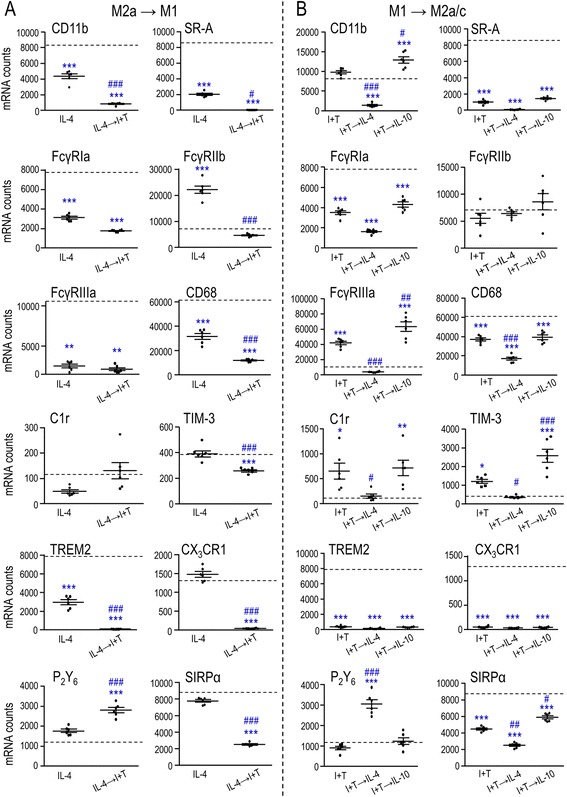
Sequential cytokine addition affects expression of phagocytosis-related molecules. **a** M2a→M1 paradigm. Microglia were treated with 20 ng/mL IL-4 for 2 h followed by 20 ng/mL IFN-γ + 50 ng/mL TNF-α (I + T) for 22 h. **b** M1→M2 paradigm. Microglia were treated with I + T for 2 h followed by either IL-4 (M1→M2a) or 20 ng/mL IL-10 (M1→M2c) for 22 h. Data are expressed as mRNA counts in 200 ng total RNA (mean ± SEM, *n* = 6 individual cultures) and were analyzed by one-way ANOVA with Tukey’s post hoc test. The *dashed lines* indicate expression levels in unstimulated (control) microglia. The comparisons are as follows: *asterisk* differences from control microglia, *number sign* effects of the second stimulus on the first stimulus. *One symbol p* < 0.05, *two symbols p* < 0.01, *three symbols p* < 0.001

**Fig. 6:**
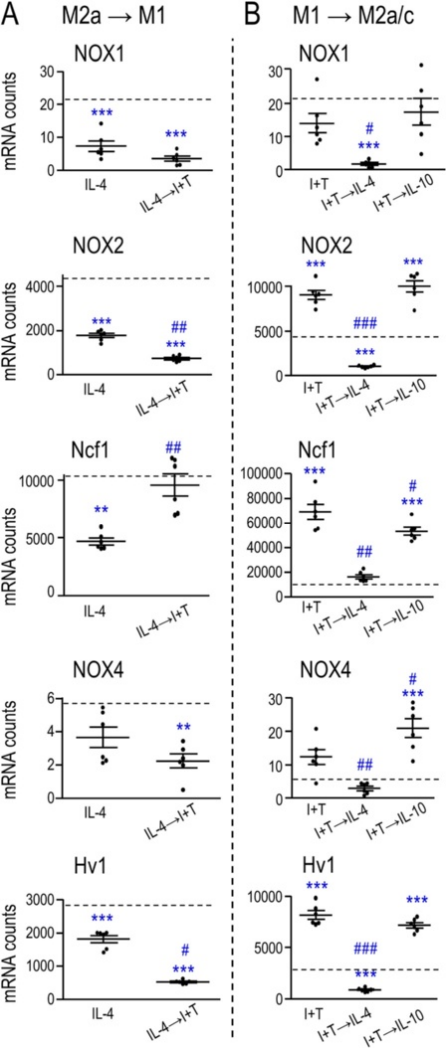
Sequential cytokine addition affects expression of ROS-associated genes. **a** M2a→M1. Microglia were treated with 20 ng/mL IL-4 for 2 h followed by 20 ng/mL IFN-γ + 50 ng/mL TNF-α (I + T) for 22 h. **b** M1→M2. Microglia were treated with I + T for 2 h followed by either IL-4 (M1→M2a) or 20 ng/mL IL-10 (M1→M2c) for 22 h. Data are expressed as mRNA counts in 200 ng total RNA (mean ± SEM, *n* = 6 individual cultures) and were analyzed by one-way ANOVA with Tukey’s post hoc test. The *dashed lines* indicate expression levels in unstimulated (control) microglia. The comparisons are as follows: *asterisk* differences from control microglia, *number sign* effects of a secondary stimulus on the first stimulus. *One symbol p* < 0.05, *two symbols p* < 0.01, *three symbols p* < 0.001

Next, we examined the same phagocytosis-related (Fig. 5) and ROS-related molecules (Fig. 6) as in Figs. 2 and 3. ***(i)*** In the M2a→M1 stimulus paradigm (Fig. 5a), there was a dampened response compared with IL-4 alone. Eight out of 12 phagocytosis-related receptors decreased, including 6 stimulatory receptors (CD11b, SR-A, CD68, TREM2, CX_3_CR1, TIM-3) and 2 inhibitory receptors (FcγRIIb, SIRPα). Three stimulatory receptors were unchanged (FcγRIa, FcγRIIIa, C1r), and only P_2_Y_6_ increased. For ROS-related molecules, Ncf1 increased but the others generally decreased (Fig. 6a). ***(ii)*** In the M1→M2a paradigm (Fig. 5b), compared with I + T alone, there was a dampened response of several stimulatory receptors (CD11b, FcγRIIIa, CD68, C1r, TIM-3), the inhibitory receptor, SIRPα, and all ROS-related genes (Fig. 6b). Genes that were unchanged were the stimulatory receptors, SR-A, FcγRIa, and TREM2, and the inhibitory receptor, FcγRIIb. Again, only P_2_Y_6_ was increased. ***(iii)*** The M1→M2c paradigm did not dampen responses compared with I + T alone, except for a small decrease in Ncf1 (Figs. 5b and 6b). Instead, there were increases in 5/17 phagocytosis- and ROS-related molecules: NOX4, the stimulatory receptors, CD11b, FcγRIIIa, and TIM-3, and the inhibitory receptor, SIRPα. Together, these results indicate substantial repolarization in the M2a→M1 and M1→M2a paradigms but not the M1→M2c paradigm.

**Fig. 7:**
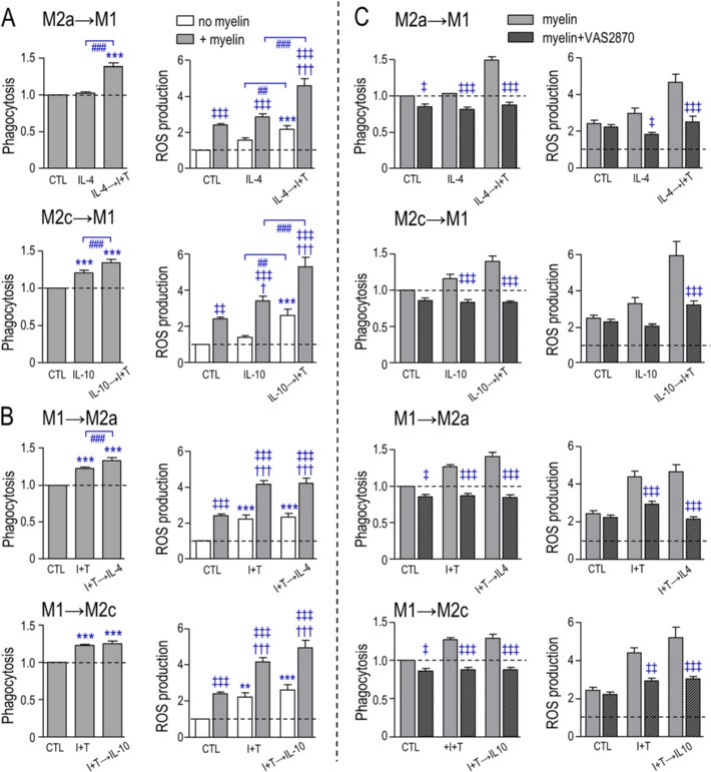
Sequential cytokine addition affects myelin phagocytosis and NOX-mediated ROS production. Data are presented as mean ± SEM; *n* = 12 (**a**, **b**), *n* = 6 (**c**). Data were normalized to unstimulated (*CTL*) microglia (indicated by *dashed lines*) in the presence of myelin (for phagocytosis) or without myelin (for ROS production). **a** M2→M1. Microglia were first stimulated with 20 ng/mL IL-4 (M2a) or 20 ng/mL IL-10 (M2c) for 2 h and then with 20 ng/mL IFN-γ + 50 ng/mL TNF-α (I + T; M1) for an additional 22 h. **b** M1→M2. I + T was added for 2 h, followed by IL-4 (M1→M2a) or IL-10 (M1→M2c) for a further 22 h. For **a** and **b**, DiI-labeled myelin (25 μg/mL) was added to cultures 24 h after adding the first cytokine, and both phagocytosis and intracellular ROS levels were assessed 6 h later (as in Figs. 2b,c and 3b,c). **c** Effect of NOX inhibition on myelin phagocytosis and ROS production in microglia that were stimulated as in **a** and **b**. Microglia were incubated with 25 μg/mL myelin for 6 h, with or without the pan-NOX inhibitor, 5 μM VAS2870. Results were analyzed by a one-way ANOVA (for phagocytosis in **a**) or a two-way ANOVA (for ROS and phagocytosis in **c**) with Bonferroni’s post hoc test. The comparisons are as follows. *Asterisk* Differences from unstimulated (CTL) microglia. *Dagger sign CTL* versus different activation states in the presence of myelin. *Number sign* Differences within (or among) different activation states. *Double dagger sign* Effects of myelin (**a**, **b**) or VAS2870 (**c**) within or between activation states. *One symbol p* < 0.05, *two symbols p* < 0.01, *three symbols p* < 0.001

The observed changes in expression of phagocytosis- and ROS-related genes led us to directly examine myelin phagocytosis and ROS production following sequential treatments with M2 and M1 stimuli. ***(i)*** M2→M1: When IL-4 (M2a)- or IL-10 (M2c)-treated microglia were subsequently treated with I + T, both phagocytosis and ROS production increased compared with IL-4 or IL-10 alone (Fig. 7a), which suggests that some re-polarization occurred. ***(ii)*** M1→M2: In the reverse paradigm (I + T, then IL-4 or IL-10), phagocytosis was slightly increased by IL-4 (M1→M2a) but not by IL-10 (M1→M2c). ROS production was unchanged compared with I + T alone (Fig. 7b). ***(iii)*** Myelin effects: Without myelin, I + T increased ROS and subsequently adding IL-4 or IL-10 had no further effect; whereas, adding I + T after IL-4 or IL-10 increased ROS production. Adding myelin always increased ROS production compared with control cells (no treatment, no myelin). In the presence of myelin, there was some functional re-polarization from M2a/c→M1 (increased phagocytosis and ROS production). In contrast, compared with I + T alone, ROS production was not altered by M1→M2a (which slightly increased phagocytosis) nor M1→M2c (which did not).

NOX enzymes were involved in both myelin phagocytosis and ROS production under almost all conditions (Fig. 7c). That is, the NOX inhibitor, VAS2870, decreased myelin phagocytosis under all conditions and decreased ROS production in all stimulated cells (but not in control cells).

### Expression of K^+^ and CRAC channels and their contributions to myelin phagocytosis and ROS production

**Fig. 8:**
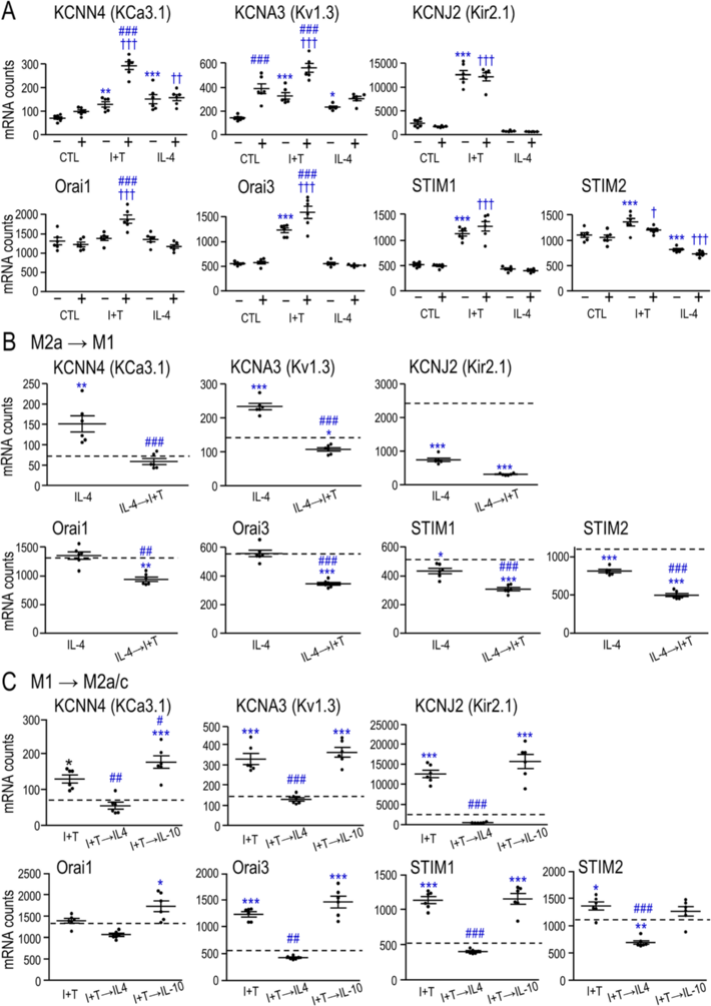
Transcript expression of K^+^ channels and SOCE-related genes is affected by the microglial activation state and myelin phagocytosis. **a** Single cytokines with or without myelin. Microglia were stimulated for 24 h with 20 ng/mL IFN-γ + 50 ng/mL TNF-α (I + T) or 20 ng/mL IL-4, and then treated with 25 μg/mL myelin for 6 h. **b** M2a→M1 paradigm. Microglia were treated with 20 ng/mL IL-4 for 2 h followed by I + T for 22 h. **c** M1→M2 paradigm. Cells were treated with I + T for 2 h followed by IL-4 (M1→M2a) or 20 ng/mL IL-10 (M1→M2c) for 22 h. All data are expressed as mRNA counts in 200 ng total RNA (mean ± SEM) for six individual cultures. Data were analyzed by one-way ANOVA with Tukey’s post hoc test. The *dashed lines* in **b** and **c** indicate expression levels in unstimulated (control) microglia. Comparisons are as follows: *asterisk* differences from control microglia, *dagger sign* CTL versus different activation states in the presence of myelin, *number sign* effects of myelin within a particular activation state (**a**) or effects of a second stimulus on the first stimulus (**b** and **c**). *One symbol p* < 0.05, *two symbols p* < 0.01, *three symbols p* < 0.001

Rat microglia express several K^+^ channels, including KCa3.1, Kv1.3, and Kir2.1, as well as store-operated Ca^2+^ entry (SOCE) channels. Pore-forming Orai1 and accessory STIM1 subunits form CRAC channels, while Orai3 and STIM2 are also involved in SOCE [64]. We first examined whether expression of these channels differs in M1 and M2 microglial activation states and whether it is altered by myelin phagocytosis (Fig. 8a). ***(i)*** In unstimulated (control) cells, the only change evoked by myelin was an increase in Kv1.3 expression. ***(ii)*** I + T (M1) stimulation increased expression of all the genes examined (except Orai1), i.e., KCa3.1 (1.8 fold), Kv1.3 (2.3 fold), Kir2.1 (5.2 fold), Orai3 (2.2 fold), STIM1 (2.2 fold), and STIM2 (1.2 fold) (Fig. 8a). In I + T treated cells, myelin phagocytosis increased expression of KCa3.1, Kv1.3, Orai1, and Orai3. ***(iii)*** IL-4 (M2a) stimulation increased expression of KCa3.1 (2.1 fold) and Kv1.3 (1.6 fold) and decreased STIM2 (by 26 %). It did not affect Kir2.1, Orai1, Orai3, or STIM1. Myelin phagocytosis did not alter any of these genes. ***(iv)*** In the M2a→M1 paradigm (IL-4 then I + T; Fig. 8b), all the genes decreased to the control level or lower, except Kir2.1. ***(v)*** In the M1→M2a paradigm (I + T then IL-4; Fig. 8c), all the genes (except Orai1) decreased compared with I + T and were then at or below the control level. ***(vi)*** In the M1→M2c paradigm (I + T then IL-10; Fig. 8c), IL-10 did not exert the same effects as IL-4. KCa3.1 was slightly elevated compared with I + T but no other genes were affected.

**Fig. 9:**
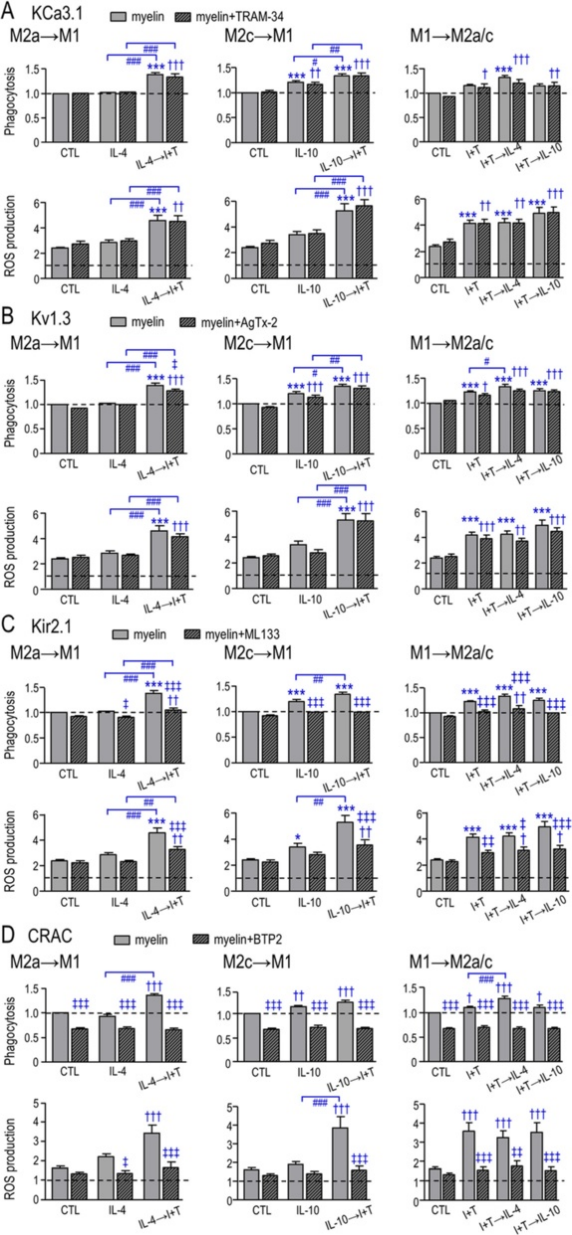
Roles of K^+^ and CRAC channels in myelin phagocytosis and ROS production. Microglia were stimulated for 24 h (as in Fig. 8), and then a channel blocker was added with or without myelin for a further 6 h. The *panels* show single stimuli, as well as M2a→M1 (*left*), M2c→M1 (*middle*), and both M1→M2a and M1→M2c (*right*). **a** KCa3.1 inhibition using 1 μM TRAM-34. **b** Kv1.3 inhibition using 5 nM Agitoxin-2 (AgTx-2). **c** Kir2.1 inhibition using 20 μM ML133. **d** CRAC inhibition using 10 μM BTP2. Graphical data are expressed as mean ± SEM (*n* = 6 individual cultures) and were analyzed by a two-way ANOVA with Bonferroni’s post hoc test. The *dashed lines* indicate levels in unstimulated microglia with myelin. Comparisons are as follows: *asterisk* differences from control microglia, *dagger sign* CTL versus activation states in the presence of a channel inhibitor, *number sign* effect of a second stimulus on activated microglia, *double dagger sign* effect of a channel inhibitor within treatment group. *One symbol p* < 0.05, *two symbols* p < 0.01, *three symbols p* < 0.001

A panel of ion channel blockers was used to assess their involvement in myelin phagocytosis and ROS production. Blocking KCa3.1 (Fig. 9a) or Kv1.3 (Fig. 9b) did not alter myelin phagocytosis or ROS production under any activation state tested. Blocking Kir2.1 had no effect on control cells (Fig. 9c) but it abolished the increase in phagocytosis under all activation paradigms. Most striking was that whenever I + T was present, Kir2.1 block reduced the myelin-induced increases in ROS production. CRAC channel inhibition greatly decreased myelin phagocytosis under all activation paradigms (Fig. 9d). ROS production was also reduced, except in control and IL-10-treated cells, where the CRAC blocker showed a trend toward a decrease that did not reach statistical significance. However, the myelin-stimulated ROS component was relatively small under these conditions, and there was less likelihood of seeing a blocker effect. There appear to be multiple components of total ROS production: a background component (without myelin) and one that was stimulated by myelin phagocytosis under all conditions (Fig. 2c), as well as a component that was not reduced by the NOX inhibitor (Fig. 3c). In principle, channel blockers could reduce ROS by affecting any component. Overall, expression of all four ion channels was affected by the activation state, but only Kir2.1 and CRAC channels were involved in myelin phagocytosis and the consequent respiratory burst.

## Discussion

This study used gene profiling and functional analyses to examine relationships between microglial activation states, myelin phagocytosis and ROS production, and the role of selected ion channels in these processes. Because the treatments and outcomes examined were complex, for clarity in comparing with previous studies, the salient findings will be discussed under four topics: ***(1)*** outcomes of single stimuli used to polarize microglia to different activation states, ***(2)*** effects of myelin phagocytosis on their activation state, ***(3)*** attempts to re-polarize microglial activation, and ***(4)*** expression and contributions of the ion channels in different activation states.

### Effects of single stimuli

The first step was to validate the procedures used to stimulate primary rat microglia. In agreement with well-known markers [17–20], we found that IFN-γ combined with TNF-α (I + T) induced a pro-inflammatory M1-like state, while IL-4 induced an anti-inflammatory (M2a) state. Thus, the cytokine concentrations we used were effective in changing the molecular inflammatory profile, as measured at 24 h.

It is expected that phagocytosis by activated microglia will depend on the target, whether it is opsonized, and which phagocytosis-related receptors are engaged. For instance, in the damaged CNS, if extravasation of complement or antibodies occurs, this can engage CR3 and Fcγ receptors, respectively. Furthermore, under in vitro conditions, if complement is present (i.e., if serum is not heat-inactivated), this can greatly promote myelin phagocytosis [32]. We previously showed that unstimulated rat microglia can phagocytose polymer beads, yeast, and *Escherichiacoli* bacteria [11, 29, 65] and that *E. coli* phagocytosis was robustly increased by LPS and IFN-γ, separately or in combination [29]. Here, untreated rat microglia robustly phagocytosed myelin, and this was modestly increased by I + T (M1) and IL-10 (M2c), but not by IL-4 (M2a). Our results are entirely consistent with an earlier study of rat microglia, in which stimulation with mouse recombinant IFN-γ, TNF-α, or IL-10 (note species mismatch) increased myelin phagocytosis, but IL-4 did not [22]. In contrast, IL-4 or IL-13 stimulation increased phagocytosis of myelin by human microglia [23] and of apoptotic cells by rat microglia [66].

We next asked whether there were changes in expression of specific phagocytosis-related receptors in different activation states. ***(i)*** For M1 stimulation, previous reports are inconsistent and possibly species dependent. Some changes seen in rat microglia are expected to increase their phagocytic capacity. Our earlier study found that LPS increased FcγRIa and FcγRIIIa, while phagocytosis of *E. coli* increased CR3 and SR-A [29]. The stimulatory phagocytic receptor, FcγRIIIa, is often used as an M1 marker [67, 68]. We found that I + T (M1) increased FcγRIIIa, as well as TIM-3, which promotes phagocytosis of apoptotic neurons [51]. Other changes might dampen the phagocytic capacity, but again, there are possible species differences, e.g., LPS reduced FcγRIIIa in murine microglia [21]. The TREM2 receptor aids in target internalization by microglia [69, 70]. We found that I + T dramatically decreased TREM2 in rat microglia; however, LPS increased it in murine microglia [71]. Fractalkine (CX_3_CL1) is an important chemotactic signal released by apoptotic cells [72] but in rat microglia, LPS decreased its receptor, CX_3_CR1 [73], as did I + T in the present study. Based on the changes we observed with M1 stimulation (and lack of changes in FcγRIIb, CD11b, P_2_Y_6_), we suggest that the most likely contributors to increased myelin phagocytosis were the reduced inhibitory SIRPα signal and a known increase in the ability of CR3 to bind targets under pro-inflammatory conditions [74]. Despite the increase in FcγRIIIa, it is not likely involved. It binds to the Fc component of antibodies but antibody-mediated opsonization of the myelin debris should not occur because the culture medium contained heat-inactivated serum. ***(ii)*** For M2 stimulation, published data on phagocytosis-related receptors are very limited and, again, there might be species differences. For rat microglia, we found that IL-4 treatment (M2a) increased expression of the inhibitory receptor, FcγRIIb, and substantially decreased CD11b, SR-A, CD68, and TREM2 (no changes in SIRPα, TIM-3). For murine microglia, IL-4 did not change FcγRIIb (CD32) expression [21]. Surprisingly, CD68 expression decreased in both M1 and M2a microglia and after myelin phagocytosis by unstimulated cells. CD68 is commonly used to identify activated, phagocytic microglia [7, 42–44]. Together, these results suggest that changes in receptor expression are not reliable predictors of the degree of myelin phagocytosis. Instead, protein levels and modulation might be more important. For instance, CR3 can potentiate or inhibit myelin phagocytosis depending on its conformation [32]. Although the changes we observed suggest a less phagocytic phenotype in the M2a state, myelin phagocytosis was comparable to untreated microglia. Of course, effects on phagocytosis of other targets after CNS damage (e.g., apoptotic neurons, cell debris, infiltrating blood cells) might differ by involving different receptors.

Phagocytosis is associated with elevated NOX-mediated ROS production to help kill engulfed pathogens [4, 54]. It was previously reported that untreated rat microglia robustly express the NOX2 isoform, with much lower NOX1 and NOX4 levels, and undetectable NOX3 [53]. Our results confirm this pattern and extend it to the M1 and M2a states. ***(i)*** Increased ROS production is a hallmark of M1 activation (reviewed in [20]), and we found that it was increased in I + T-treated microglia. NOX2 was likely responsible because expression of both NOX2 and its regulatory subunit, Ncf1, were increased to very high levels, while NOX1 and NOX4 remained at very low levels. ***(ii)*** IL-4 slightly increased ROS production while IL-10 had no effect, and this is consistent with our recent report [34]. IL-4 decreased expression of NOX2 and did not change Ncf1; however, both remained at moderate levels and could account for the ROS production.

### Effects of myelin phagocytosis

There is some evidence that myelin phagocytosis can affect the M1 activation state. Effects are potentially time dependent, and negative self-regulation might protect the cells from “overeating” during extended exposures to targets [2]. When murine microglia were stimulated with IFN-γ or LPS, a short exposure to myelin (≤6 h) exacerbated the pro-inflammatory response [10], while longer exposures (16–24 h) dampened this response [10, 75]. Using the short exposure time (6 h), which was sufficient for optimal myelin uptake (see the “Methods” section); we found little effect on the molecular profile of unstimulated rat microglia. In contrast, myelin increased expression of pro-inflammatory cytokines in M1 (I + T-treated) rat microglia. This is consistent with the previous short exposure study [10]. In IL-4-treated cells, myelin reduced several M2a-associated molecules. These results suggest that myelin can skew activated rat microglia toward a pro-inflammatory state.

Because levels of phagocytosis-related receptors were not well predicted by the microglial activation state (above); it was important to ask whether they were altered by exposure to myelin debris. In unstimulated microglia, myelin slightly decreased SIRPα and considerably increased CX_3_CR1 and P_2_Y_6_, which are all expected to promote phagocytosis, especially of apoptotic cells. ***(i)*** In I + T-treated (M1) cells, myelin did not alter expression of receptors known to be involved in myelin phagocytosis (CD11b, SR-A, TREM2, CX_3_CR1, SIRPα). Instead, it increased P_2_Y_6_, FcγRIIIa, C1r, and TIM-3 and slightly decreased FcγRIIb, changes that could promote phagocytosis of other targets. ***(ii)*** In IL-4-treated (M2a) cells, myelin greatly increased CX_3_CR1 and slightly increased P_2_Y_6_. CD68 was slightly decreased, and CD11b or SR-A were unchanged. Interestingly, CX_3_CR1 [76] and P_2_Y_6_ [7] promote microglial migration toward damaged cells, and we previously found that M2a-activated rat microglia migrate better [25]. Thus, exposure to myelin debris might further potentiate the migratory capacity of M2a-activated microglia.

Myelin phagocytosis increases ROS production by unstimulated microglia [10, 22]. We confirmed this and showed that the myelin-evoked ROS production required NOX activity under all activation conditions tested (I + T, IL-4, IL-10). Myelin did not affect expression of NOX enzymes, but it increased expression of the proton channel, Hv1, which could contribute to the increased ROS production seen in unstimulated and M1-activated cells. Moreover, myelin binding to Mac1 (CD11b) can activate NOX2 and promote ROS generation [77]. Interestingly, inhibiting NOX activity reduced myelin phagocytosis under all activation conditions tested. While we do not know the mechanism, an earlier study of rat macrophages suggested that NOX-mediated ROS production promotes signaling mechanisms involved in myelin phagocytosis [78].

### Sequential cytokine stimulation

After acute CNS injury, the inflammatory milieu changes over time [79, 80]. However, whether microglial activation states are functionally plastic is poorly understood. It is important to determine if, once polarized, they can respond to new signals. Based on the few studies that have addressed time-dependent changes in the overall inflammatory state in vivo, the outcome might depend on the type of injury. In the cuprizone-induced de-myelination model, an overall M1 state gave way to an M2a phenotype at the time of re-myelination [68]. In the first week after intracerebral hemorrhage, we observed concurrent elevation of pro- and anti-inflammatory mediators [81, 82]. However, after cerebral ischemia or traumatic brain injury, murine microglia exhibited an early M2 state, followed by M1 [67, 83]. An in vitro study of rat microglia found that adding IL-4 (M2a) before LPS (M1) decreased expression of the M1-associated molecules, COX-2, iNOS, and TNF-α compared with LPS alone [84]. Similarly, in mixed rat glial cell cultures, simultaneous addition of LPS and IL-4 (or IL-10; M2c) reduced IL-6, TNF-α, and NO production, compared with LPS alone [85]. Both studies assessed pro-inflammatory mediators only. For murine microglia, when LPS was followed by IL-4, NOS2 and COX-2 expression decreased, while CD206 (MRC1) and Arginase 1 (M2a markers) increased compared with LPS alone [21, 86]. The present study greatly extends these previous reports.

We examined effects of sequential addition of M1- and M2-inducing cytokines on the inflammatory profile, expression of phagocytosis-related receptors and ROS-related molecules, and on myelin phagocytosis and consequent ROS production. We employed four sequential treatment paradigms that address the possibility that the cytokine profile changes after injury or disease. The most convincing re-polarization of the inflammatory state was between I + T and IL-4 treatments, applied in both sequences. ***(i)*** M1→M2a paradigm: In I + T-primed microglia, adding IL-4 dampened the pro-inflammatory profile (NOS2, TNF-α, IL-6, COX-2) and increased M2a markers (MRC1, c-myc, CCL22). This is entirely consistent with previous studies using LPS and IL-4 (cited above). The relationship of phagocytosis and ROS production to expression of receptors and enzymes and to phagocytosis- and ROS-related molecules was complicated and, sometimes, unexpected. Although myelin phagocytosis was increased, several phagocytosis-promoting receptors decreased (CD11b, FcγRIIIa, CD68, C1r, TIM-3), compared with I + T alone. Among the inhibitory receptors, SIRPα decreased and FcγRIIb was unchanged. Thus, the decrease in SIRPα might have promoted phagocytosis. Despite the lack of change in ROS production, ROS-related molecules decreased (NOX enzymes, Ncf1, Hv1), suggesting that the remaining levels were sufficient. ***(ii)*** M2a→M1: In IL-4-treated cells, adding I + T skewed them toward an M1 profile. There was increased expression of most pro-inflammatory molecules (NOS2, TNF-α, COX-2), decreases in some M2 markers (MRC1, CD163), and increased myelin phagocytosis and ROS production. The outcome might depend on the exact stimulus paradigm and target type. For instance, we observed some changes in receptor expression that are expected to promote phagocytosis: an increase in P_2_Y_6_ and decreases in the inhibitory receptors, FcγRIIb and SIRPα. Most phagocytosis-promoting receptors decreased, particularly CD11b, TREM2, CD68, TIM-3, and CX_3_CR1. ***(iii)*** M1→M2c: In I + T-primed cells, adding IL-10 did not resolve the pro-inflammatory state and, surprisingly, it increased iNOS/NOS2 expression. While expression of several phagocytosis-related receptors increased (CD11b, FcγRIIIa, TIM-3), SIRPα increased slightly, while phagocytosis and ROS production were unchanged. ***(iv)*** M2c→M1: In IL-10-treated cells, adding I + T increased myelin phagocytosis and ROS production (gene changes were not examined, as explained above).

Overall, our results show malleability in re-polarization of rat microglia between M1 and M2a states. Their qualitative ability to respond to a new incoming signal was preserved but their quantitative response was often reduced. The amount of re-polarization could well depend on the experimental paradigm (cytokine concentrations, time course). For instance, although we did not observe resolution of M1 activation by IL-10, it is possible that the time course of treatment or monitoring was not optimal. In the future, in vivo spatial and temporal changes in the cytokine environment will need to be examined in each damage/disease model to further examine the re-polarization capacity of microglia.

### Expression and contributions of ion channels

Ion channels regulate numerous processes in cells that are relevant to phagocytosis, including cell volume, Ca^2+^ signaling, and cytoskeletal re-organization [87]. Microglia express a surprisingly large array of ion channels, some of which are involved in proliferation, migration, and Ca^2+^ signaling (reviewed in [42, 88]). Very little is known about roles of channels in phagocytosis by microglia; particularly in different activation states. We previously found that Cl^−^ channels regulate phagocytosis of *E. coli* by rat microglia [65]. Phagocytosis is a Ca^2+^-dependent process that involves SOCE [89, 90]. The CRAC channel is apparently the major SOCE pathway in rat microglia [33, 35, 91, 92]. Interestingly, CRAC can be activated by P_2_Y_6_ and other G protein-coupled metabotropic receptors, and we found that phagocytosis of myelin debris increased P_2_Y_6_ expression under all activation states examined. We then focused on CRAC and three K^+^ channels (KCa3.1, Kir2.1, Kv1.3) that are thought or known to regulate Ca^2+^ entry. In rat microglia, KCa3.1 and Kir2.1 regulate CRAC-mediated Ca^2+^ influx [24, 33, 35], and Kv1.3 is involved in ROS production [14, 37]. CRAC channels are comprised of a pore-forming Orai1 subunit and a Ca^2+^-sensing STIM1 subunit [64]. While other subunits are considered less important, SOCE in murine microglia might also involve STIM2 [90], and Orai3 activity can also be regulated by STIM proteins [64]. Rodent microglia express mRNA for Orai1, Orai3, STIM1, and STIM2 [89–91]. In murine microglia, Orai1, STIM1, and STIM2 contribute to SOCE and phagocytosis [89, 90]. There are few reports regarding changes in Ca^2+^ signaling and expression of relevant Ca^2+^-signaling molecules in specific activation states; and the results are somewhat inconsistent. For murine microglia, LPS (M1) increased STIM1 without affecting Orai1 or Orai3 in one study [89], but reduced STIM1 and Orai3 without affecting Orai1 or STIM2 in another [90]. Orai1 expression was not changed in either study but SOCE was reduced. The Ca^2+^ entry pathway was not determined. An earlier study of murine microglia reported that LPS rapidly elevated basal Ca^2+^ and reduced the UTP-induced rise [93]. For human microglia, M1 stimulation (GM-CSF + LPS + IFN-γ) did not affect the Ca^2+^ response to ADP; whereas, M2 activation (M-CSF + IL-4 + IL-13) increased it and this was attributed to increased P_2_Y_12_ receptor expression [94]. For rat microglia, we recently found that the CRAC-mediated Ca^2+^ rise was ~50 % lower after IL-4 treatment (M2a) but not affected by IL-10 (M2c) [35].

In unstimulated rat microglia, myelin affected expression of Kv1.3 only (increased) but channel expression was strongly affected by the microglial activation state. ***(i)*** I + T-treated (M1) cells had increased expression of Kv1.3, KCa3.1, Kir2.1, STIM1, STIM2, and Orai3. Myelin phagocytosis increased Kv1.3, KCa3.1, Orai1, and Orai3. The high STIM expression and increase in Orai1, the pore-forming subunit of CRAC, should facilitate Ca^2+^ signaling. ***(ii)*** IL-4-treated (M2a) cells had increased Kv1.3 and KCa3.1 compared with control cells. Myelin had no further effects. No Orai or STIM molecules increased but STIM2 decreased slightly. Thus, the previously observed decrease in CRAC signaling [35] is not readily explained by Orai or STIM expression. ***(iii)*** Sequential cytokine addition had complex effects on channel expression. M1→M2a: Compared with I + T stimulation alone, subsequent IL-4 addition reduced KCa3.1, Kv1.3, Kir2.1, Orai3, STIM1, and STIM2. M2a→M1: Compared with IL-4 alone, subsequent I + T addition decreased expression of KCa3.1, Kv1.3, Orai3, STIM1, and STIM2, but not Kir2.1. M1→M2c: Compared with I + T alone, subsequent addition of IL-10 only changed KCa3.1, which was increased. Overall, with respect to ion channels, rat microglia showed considerable re-polarization between M1 and M2a states, while IL-10 was ineffective.

The use of selective channel blockers showed that CRAC was important for phagocytosis under all activation conditions examined, while myelin phagocytosis and ROS production by activated microglia were both dependent on Kir2.1 (but not Kv1.3 or KCa3.1). What could account for a lack of contribution of Kv1.3 and KCa3.1 when, in principle, all routes of K^+^ flux can control the membrane potential of cells? The simplest possibility is that, despite substantial transcript expression, Kv1.3 and KCa3.1 were not active during myelin phagocytosis. We propose a scenario in which Kv1.3 and KCa3.1 are inhibited, while Kir2.1 and CRAC are facilitated. All three K^+^ channels are post-translationally regulated by signaling molecules downstream of the phagocytosis receptors, CR3 and SR-A. CR3 signaling activates Src family tyrosine kinases, phosphoinositide-3-kinase (PI3K) and phospholipase C (PLC) [74, 95]. Kv1.3 is strongly inhibited by activated Src in rat microglia [36]. Lipid phosphatases localize to phagosome cups (see reviews [55, 74]), and the lipid phosphatase, myotubularin-related protein 6 (MTMR6) regulates macropinocytosis [96], which uses similar machinery to phagocytosis [97]. KCa3.1 is strongly inhibited by MTMR6 [98]. How might Kir2.1 and CRAC channel activity be promoted? Both CR3 and SR-A signaling involve PLC [99] and PI3K [100]. PI3K generates PIP2, which stabilizes the open configuration of Kir2.1 channels [101]. PLC activity generates diacylglycerol (DAG) and inositol triphosphate (IP_3_), which depletes ER calcium stores and activates CRAC channels [55]. In addition, DAG activates protein kinase C, which can stimulate NOX enzymes and increase ROS production [54].

## Conclusions

Experimental strategies for treating acute CNS injury increasingly address inflammation, often targeting pro- or anti-inflammatory states in a generalized manner. Some preclinical studies target innate immune cells (microglia, macrophages, neutrophils) more selectively, but it is crucial to investigate potential targets in different cell activation states. Further specificity in target selection will be necessary because some products and functions can be either detrimental or beneficial, as is the case for phagocytosis. In CNS disease and injury states where white matter is damaged, efficient re-myelination requires that microglia remove myelin debris from affected axons. Therefore, treatment strategies should preserve microglial phagocytosis while reducing harmful inflammatory responses.

Here, we investigated numerous molecular and functional changes in rat microglia skewed to different M1 and M2 activation states. Our results illustrate several complex outcomes that should be considered in pursuing strategies to target microglia. For instance, the M1 state increased phagocytosis of myelin debris, which could aid in tissue repair, but NOX-mediated ROS production was also increased, which might damage bystander cells. Myelin phagocytosis exacerbated M1 activation, decreased M2 activation, and evoked more NOX-mediated ROS production, suggesting a positive-feedback network that might increase damage. Using sequential cytokine addition to model changing activation cues after acute CNS injury, we asked whether microglia can be re-polarized from one activation state to another. Qualitative molecular re-polarization was seen between M1 and M2a states with the paradigms used, which supports attempts to re-program the inflammatory response in vivo. Moreover, because both M1→M2 and M2→M1 paradigms increased myelin phagocytosis, it might be possible to maintain efficient debris clearance while therapeutically altering inflammatory mediators to a less toxic mix.

Ion channels are increasingly proposed as molecular targets for controlling CNS inflammation. This study contributes information about channel expression and functional contributions that should be taken into account. While Kv1.3 and KCa3.1 expression were affected by the microglial activation state, neither channel contributed to myelin phagocytosis. Thus, Kv1.3 and KCa3.1 blockers might be useful for reducing inflammation without preventing beneficial debris clearance. On the other hand, Kir2.1 and CRAC channels facilitated myelin phagocytosis in all activated states, suggesting that stimulating their activity might aid in debris clearance. However, our results also suggest that facilitating these channels will also increase ROS production whenever an M1 stimulus is present, and this could damage bystander cells.

This study greatly extends our knowledge by examining effects of single versus sequential addition of M1- and M2-inducing cytokines. Results on myelin phagocytosis and consequent ROS production are most relevant to diseases involving white matter damage, such as spinal cord injury, stroke, hemorrhage, brain trauma, MS, and ALS. However, results concerning the inflammatory profile, expression of phagocytosis-related receptors, ROS-related molecules, and ion channels will be broadly applicable to CNS injury and disease.

## Abbreviations

CD: cluster of differentiation
CNS: central nervous system
CR3: complement receptor 3
CRAC: calcium release activated calcium
DAG: diacylglycerol
DCF: dichlorodihydrofluorescein
DiI: 1,1′-dioctadecyl-3,3,3′,3′-tetramethylindocarbocyanine perchlorate
FBS: fetal bovine serum
HEPES: 4-(2-hydroxyethyl)-1-piperazineethanesulfonic acid
IFN-γ: interferon gamma
IL: interleukin
IP_3_: inositol trisphosphate
LAMP-1: lysosome-associated membrane glycoprotein-1
LPS: lipopolysaccharide
MEM: minimum essential medium
MTMR6: myotubularin related protein 6
NADPH: nicotinamide adenine dinucleotide phosphate
NOX: NADPH oxidase
PI3K: phosphoinositide-3-kinase
PIP2: phosphotidylinositol bisphosphate
PLC: phospholipase C
PMA: phorbol myristate acetate
RNA: ribonucleic acid
ROS: reactive oxygen species
RT-PCR: reverse transcription-polymerase chain reaction
SIRPα: signal regulatory protein alpha
SOCE: store operated calcium entry
SR-A: scavenger receptor-A
STIM: stromal interaction molecule
TIM-3: T-cell immunoglobulin and mucin-domain containing 3
TNF-α: tumor necrosis factor alpha
TREM2: triggering receptor expressed on myeloid cells 2
